# Microarray Я US: a user-friendly graphical interface to Bioconductor tools that enables accurate microarray data analysis and expedites comprehensive functional analysis of microarray results

**DOI:** 10.1186/1756-0500-5-282

**Published:** 2012-06-08

**Authors:** Yilin Dai, Ling Guo, Meng Li, Yi-Bu Chen

**Affiliations:** 1 Department of Mathematical Sciences, Michigan Technological University, 1400 Townsend Drive, Houghton, MI, 49934, USA; 2 Bioinformatics Service Program, Norris Medical Library, University of Southern California, 2003 Zonal Ave, Los Angeles, CA, 91007, USA

**Keywords:** Microarray data analysis, Gene expression, Probe reannotation

## Abstract

**Background:**

Microarray data analysis presents a significant challenge to researchers who are unable to use the powerful Bioconductor and its numerous tools due to their lack of knowledge of R language. Among the few existing software programs that offer a graphic user interface to Bioconductor packages, none have implemented a comprehensive strategy to address the accuracy and reliability issue of microarray data analysis due to the well known probe design problems associated with many widely used microarray chips. There is also a lack of tools that would expedite the functional analysis of microarray results.

**Findings:**

We present Microarray Я US, an R-based graphical user interface that implements over a dozen popular Bioconductor packages to offer researchers a streamlined workflow for routine differential microarray expression data analysis without the need to learn R language. In order to enable a more accurate analysis and interpretation of microarray data, we incorporated the latest custom probe re-definition and re-annotation for Affymetrix and Illumina chips. A versatile microarray results output utility tool was also implemented for easy and fast generation of input files for over 20 of the most widely used functional analysis software programs.

**Conclusion:**

Coupled with a well-designed user interface, Microarray Я US leverages cutting edge Bioconductor packages for researchers with no knowledge in R language. It also enables a more reliable and accurate microarray data analysis and expedites downstream functional analysis of microarray results.

## Findings

### Background

Microarray technology has been widely used for global gene expression profiling. Based on the major public microarray data repositories such as NCBI GEO (http://www.ncbi.nlm.nih.gov/geo/) and ArrayExpress (http://www.ebi.ac.uk/arrayexpress/), the overwhelming majority of microarray studies were performed on Affymetrix GeneChips and Illumina BeadArrays, with human, mouse and rat being the most common model organisms. Finding differentially expressed genes (DEG) under various experimental conditions is the primary goal of these studies.

With hundreds of published packages, the R-based statistical platform Bioconductor [1] is a major solution for microarray data analysis. However, the command-line driven Bioconductor and its packages may prove to be inconvenient to use for experienced users dealing with multiple-step analysis, and virtually inaccessible for users with no solid knowledge about R. Graphical user interfaces (GUI) for Bioconductor packages have been developed to enable biology researchers to use cutting-edge algorithms without the need of learning R, notably, affylmGUI for Affymetrix data analysis [2] and oneChannelGUI for both Affymetrix and Illumina data analysis [3]. Web-based software such as WebArray [4] and CARMAweb [5] were also developed to offer GUI to Bioconductor packages. Many of these software have been actively expanded since their initial releases, but as more and more functionalities are added, they are increasingly cumbersome to learn and use, especially for those who are mostly interested in differential expression analysis.

A shortfall common to all these software is that they generally do not systematically address the probe design problems associated with many microarray chips. The Affymetrix 3’IVT GeneChips have been the most popular platform for global gene expression analysis in the past decade. They consist of probe sets containing 11–20 pairs of 25mer probe targeting a gene or transcript. While designed with the most complete information available at the time, the tremendous progress in genome sequencing and annotation in recent years has rendered an increasing number of existing probe sets outdated. Several studies [6-8] indicated that a substantial percentage (30-70%) of Affymetrix probe sets contain at least one probe that is either non-unique, no-target, mis-targeted, or overlapping with known SNPs in the central region. Besides adding noise, these problematic probes also affect the accuracy of expression value estimation [9,10] and references therein]. Furthermore, these studies have also shown that the annotations of a significant portion of the probe sets are either outdated or incorrect based on the latest genomic knowledge. The updated Affymetrix probe set annotation not only benefits our understanding of microarray results, but also improves the cross-platform reproducibility of microarray experiments [11,12]. While the chip design philosophy is different from Affymetrix’s GeneChips, Illumina’s BeadArrays have similar problems in terms of problematic probes and outdated probe annotations [13,14].

### Implementation

**Figure 1:**
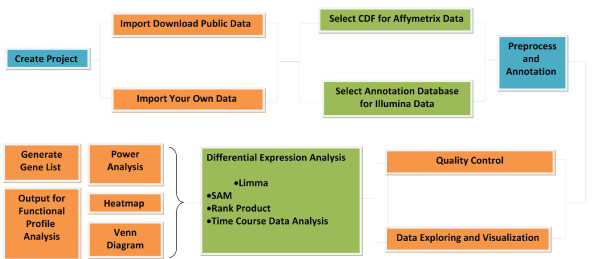
**Typical Microarray Я US workflow.** Microarray Я US provides a streamlined workflow for a typical differential gene expression analysis task.

**Figure 2:**
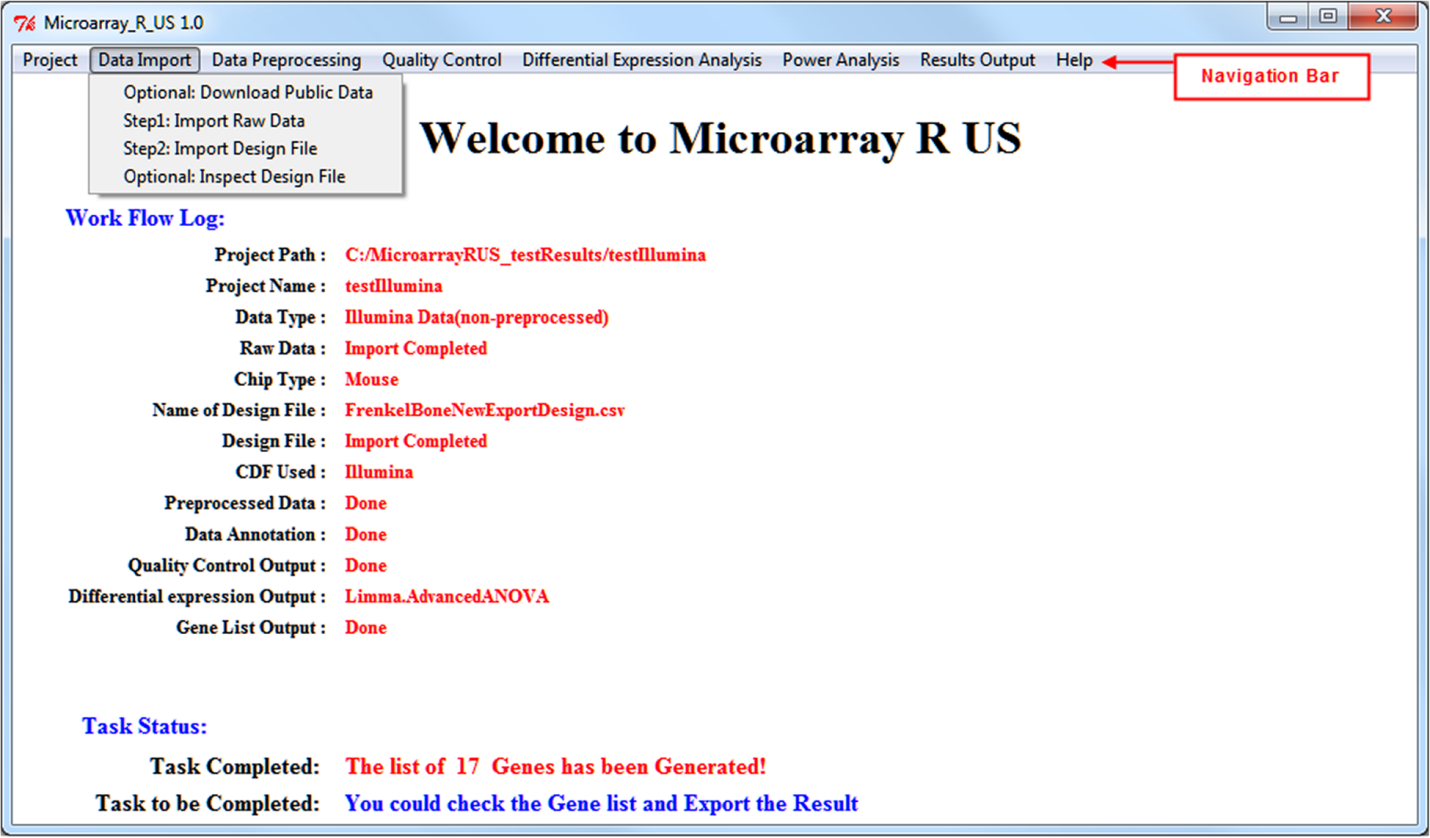
**Microarray Я US console.** Microarray Я US features a linear step-wise workflow for analyzing microarray raw data. When using the Microarray Я US, users can simply follow the workflow by going through the Navigation Bar from left to right. Major analysis steps are also clearly marked in the **Task Status** section. **Task to be Completed** directs users to the next task in the workflow.

Written in R, Microarray Я US was a cleanly designed GUI specifically for users who have no knowledge in R. It provides a streamlined workflow for analyzing expression microarray data (Figure 1). The program console consists of a top menu bar, as well as a Work Flow Log and a Task Status to allow users to easily perform and track the status of their data analysis (Figure 2). For information on all the Bioconductor packages implemented in this software as well as their publications, refer to Additional file 1. List of the implemented Bioconductor packages.

## Results and discussion

Microarray Я US was developed to not only provide a simple and streamlined workflow to researchers who are mainly interested in a fast differential gene expression analysis, but also to improve the accuracy and reliability of the analysis, as well as expedite downstream functional analysis of the microarray results. In addition to many carefully planned design characteristics aimed at enhancing its usability, Microarray Я US provides the following unique features:

### Support custom chip description files (CDF) for major 3’IVT affymetrix GeneChips and probe re-annotation for major affymetrix GeneChips and illumina BeadArrays

To enable researchers to take advantage of the latest research on probe set re-definition and re-annotation, we implemented the custom CDF and probe set re-annotation by Dai et al. (2005) and by Risueno et al. (2010) for Affymetrix GeneChips and probe re-annotation by Du et al. (2008) and Barbosa-Morais et al. (2009) for Illumina BeadArrays. To mitigate the undesirable consequences that arose from the aforementioned microarray probe design problems, Dai et al. (2005) and Risueno et al. (2010) used the latest genome/transcriptome sequences to perform strict probe re-alignment and mapping and discarded 30-60% of the original Affymetrix probes that were problematic. The remaining probes were re-defined into new probe sets (in the form of custom CDF) and re-annotated with the latest genomic annotation. An independent evaluation of Dai et al.’s study concluded that the updated probe set definitions resulted in significant improvement of both precision and accuracy of expression level analysis [15]. For Illumina arrays, Du et al. (2008) eliminated up to 30% of original probes without a unique and perfect match to a single Entrez gene by mapping probe sequences against the latest corresponding RefSeq sequences and re-annotated the remaining probes with the latest genomic annotations. Barbosa-Morais et al. (2009) also re-defined the probes against the latest genome and transcriptome but used less strict rules for excluding uninformative probes.

To our knowledge, Microarray Я US is the only microarray software that implements multiple custom CDF (Affymetrix) and probe set/probe re-annotation (Affymetrix and Illumina) for a more reliable gene expression analysis.

### Quick generation of input files for comprehensive functional analysis of microarray results

The statistical analysis of microarray raw data often results in lists of hundreds of DEG. Understanding the underlying mechanisms and functional ramification of such expression changes is becoming the most important and daunting task of ‘Omics research. In the last decade, several hundred bioinformatics tools have been developed for biological interpretation of large gene lists at a systems biology level [reviewed in 16]. A comprehensive functional analysis of microarray results commonly requires the use of multiple tools, as they differ in underlying statistical methods, annotation contents and analytical capabilities [16,17]. Different tools usually require different types/format of input files, and manually converting microarray results into these files is a very laborious task. To expedite comprehensive functional analysis, we implemented a results output utility tool that can instantly generate input files for some 20 of the most widely used commercial and open access functional analysis software (see Additional file 2. List of the supported functional analysis software). To our knowledge, Microarray Я US is the only microarray software that provides such time-saving functionality.

### Microarray Я US key functionalities

#### Data import

Microarray Я US supports major 3’IVT Affymetrix GeneChips and Illumina BeadArrays for human, mouse and rat (see Additional file 3. List of the supported microarray data types). In addition to user data, public data from GEO and ArrayExpress can be directly downloaded within the software via the implementation of *GEOquery*, *GEOmetadb*, and *ArrayExpress*.

#### Custom CDF and probe re-annotation selections

For Affymetrix GeneChips, users are given choices of the original manufacturer and custom CDF [6, 7, Brainarray version 13], along with the corresponding probe set annotations. For Illumina BeadArrays, original manufacturer annotation and two custom re-annotation [13,14] are available.

#### Data preprocessing

For Affymetrix data, Microarray Я US offers several commonly used algorithms as implemented in RMA, gcRMA, MAS5 and dChip packages. An advanced option is also provided to allow users to select methods for background correction, PM correction, normalization, and probe set summarization. For Illumina data, the software accepts preprocessed data output from GenomeStudio and supports fully customizable preprocessing with *lumi* package for non-preprocessed data.

#### Quality control and exploratory analysis

For Affymetrix data, Microarray Я US implemented *ArrayQualityMetrics* and *QCreport*. For Illumina data, the quality control method implemented in the *lumi* package is supported.

For exploratory data analysis, Microarray Я US supports both Principle Component Analysis and hierarchical clustering analysis via the implementation of *made4* and *stats* packages.

#### Differential expression analysis

Four widely used statistical packages are implemented, including Linear Model for Microarray Data (*limma*, with advanced options for multiple fixed and random factors), Significance Analysis of Microarrays (*SAM*, both paired and unpaired), Rank Product Test (*RankProd*), and *maSigPro* (time course data).

#### Power analysis

Power analysis on sample size and detection efficiency for p value or fold changes are supported in Microarray Я US via the implementation of *ssize* package.

#### Results output

With easy to follow dialog windows, users can output a full table of statistical results or DEG lists. Visualizations of DEG lists with heatmap or Venn diagrams are also available via the implementation of *gplots* and *limma* packages.

**Figure 3:**
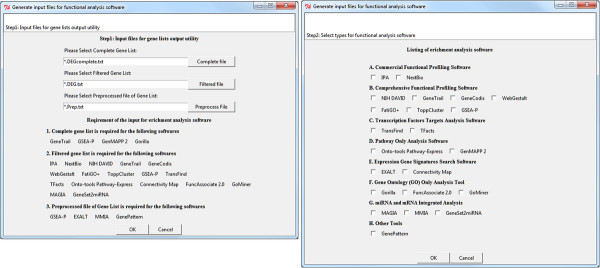
**Result Output Utility Dialog Windows.** The Result Output Utility Tool of Microarray Я US exports microarray results into input files for over 20 commonly used function analysis software with corresponding formats. This function can also be used for converting results generated with other microarray analysis software.

**Figure 4:**
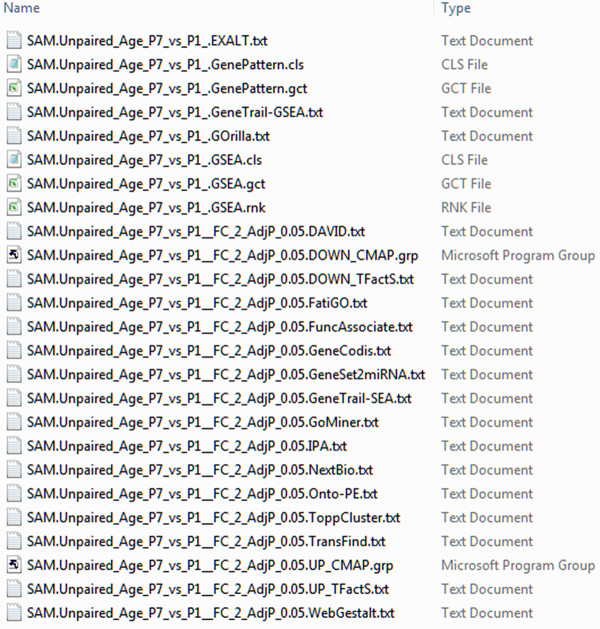
**Examples of output results files.** The statistical methods, experimental factor and the functional analysis software name are automatically embedded in the names of output files. The output files can then be directly imported into the corresponding functional analysis software.

The Gene List Output Utility can be used to instantly convert microarray results into input files for over 20 functional analysis software (Figure 3. Screenshot of the dialog windows for generating input files for functional analysis tools). It can also be used for microarray results generated from third-party microarray software with minimal reformatting. A carefully-thought default file naming schema was implemented to allow users to easily locate output files for each selected functional analysis tool (Figure 4. Examples of output results files for downstream functional analysis).

## Conclusion

A GUI to over a dozen widely used Bioconductor packages with enhanced usability, Microarray Я US provides a streamlined workflow for routine differential gene expression analysis based on Affymetrix and Illumina chips for users with no knowledge in R language. With its unique implementation of several up-to-date Affymetrix custom CDF and probe set re-annotations for both Affymetrix and Illumina platforms, this tool facilitates a more accurate and precise microarray data analysis. The versatile results output utility tool enables a fast and easy generation of input files for over 20 of the most popular functional analysis software programs.

### Availability and requirements

Microarray Я US is available for Windows (both 32 and 64 bit), Mac OS, and Linux/Unix under the Open GPL license at http://norris.usc.libguides.com/MicroarrayRUS (free registration required).

Periodic update of the custom CDF will be made when the major revisions become available.

## Abbreviations

DEG, Differentially expressed genes; GUI, Graphical user interfaces; CDF, Chip description files.

## Competing interests

The authors declare that they have no competing interests.

## Authors’ contribution

YC conceived the project, advised on software design and drafted the manuscript. YD and LG performed the designing, coding, and debugging. ML and LG prepared user documentation. ML carried out software testing, web publishing and manuscript preparation. All authors participated in manuscript revisions and have read and approved the final manuscript.

## Supplementary Material

Additional file 1 **List of the implemented Bioconductor packages.** Complete list of the implemented Bioconductor packages with brief descriptions and references. Click here for file

Additional file 2 **List of the supported functional analysis software.** Description: Complete list of the supported functional analysis software for the Gene List Output Utility Tool. Access information, methods, input file requirements, supported organisms, matching Microarray Я US output file, and other details are listed for each supported software. Click here for file

Additional file 3 **List of the supported microarray data types.** Complete list of the supported microarray chips. Click here for file

## References

[B1] Gentleman RC Carey VJ Bates DM Bolstad B Dettling M Dudoit S Ellis B Gautier L Ge Y Gentry J Bioconductor: open software development for computational biology and bioinformatics Genome biology 2004 5 R80 10.1186/gb-2004-5-10-r80 15461798 PMC545600

[B2] Wettenhall JM Simpson KM Satterley K Smyth GK affylmGUI: a graphical user interface for linear modeling of single channel microarray data Bioinformatics 2006 22 897 899 10.1093/bioinformatics/btl025 16455752

[B3] Sanges R Cordero F Calogero RA oneChannelGUI: a graphical interface to Bioconductor tools, designed for life scientists who are not familiar with R language Bioinformatics 2007 23 3406 3408 10.1093/bioinformatics/btm469 17875544

[B4] Xia X McClelland M Wang Y WebArray: an online platform for microarray data analysis BMC Bioinformatics 2005 6 306 10.1186/1471-2105-6-306 16371165 PMC1327694

[B5] Rainer J Sanchez-Cabo F Stocker G Sturn A Trajanoski Z CARMAweb: comprehensive R- and bioconductor-based web service for microarray data analysis Nucleic Acids Res 2006 34 W498 W503 10.1093/nar/gkl038 16845058 PMC1538903

[B6] Risueno A Fontanillo C Dinger ME De Las Rivas J: GATExplorer: genomic and transcriptomic explorer; mapping expression probes to gene loci, transcripts, exons and ncRNAs BMC Bioinformatics 2010 11 221 10.1186/1471-2105-11-221 20429936 PMC2875241

[B7] Dai M Wang P Boyd AD Kostov G Athey B Jones EG Bunney WE Myers RM Speed TP Akil H Evolving gene/transcript definitions significantly alter the interpretation of GeneChip data Nucleic Acids Res 2005 33 e175 10.1093/nar/gni179 16284200 PMC1283542

[B8] Ballester B Johnson N Proctor G Flicek P Consistent annotation of gene expression arrays BMC Genomics 2010 11 294 10.1186/1471-2164-11-294 20459806 PMC2894801

[B9] Gautier L Cope L Bolstad BM Irizarry RA affy–analysis of Affymetrix GeneChip data at the probe level Bioinformatics 2004 20 307 315 10.1093/bioinformatics/btg405 14960456

[B10] Nurtdinov RN Vasiliev MO Ershova AS Lossev IS Karyagina AS PLANdbAffy: probe-level annotation database for Affymetrix expression microarrays Nucleic Acids Res 2010 38 D726 D730 10.1093/nar/gkp969 19906711 PMC2808952

[B11] Carter SL Eklund AC Mecham BH Kohane IS Szallasi Z Redefinition of Affymetrix probe sets by sequence overlap with cDNA microarray probes reduces cross-platform inconsistencies in cancer-associated gene expression measurements BMC Bioinformatics 2005 6 107 10.1186/1471-2105-6-107 15850491 PMC1127107

[B12] Elo LL Lahti L Skottman H Kylaniemi M Lahesmaa R Aittokallio T Integrating probe-level expression changes across generations of Affymetrix arrays Nucleic Acids Res 2005 33 e193 10.1093/nar/gni193 16356924 PMC1316121

[B13] Barbosa-Morais NL Dunning MJ Samarajiwa SA Darot JF Ritchie ME Lynch AG Tavare S A re-annotation pipeline for Illumina BeadArrays: improving the interpretation of gene expression data Nucleic Acids Research 2010 38 e17 10.1093/nar/gkp942 19923232 PMC2817484

[B14] Du P Kibbe WA Lin SM lumi: a pipeline for processing Illumina microarray Bioinformatics 2008 24 1547 1548 10.1093/bioinformatics/btn224 18467348

[B15] Sandberg R Larsson O Improved precision and accuracy for microarrays using updated probe set definitions BMC Bioinformatics 2007 8 48 10.1186/1471-2105-8-48 17288599 PMC1805763

[B16] Huang DW Sherman BT Lempicki RA Bioinformatics enrichment tools: paths toward the comprehensive functional analysis of large gene lists Nucleic Acids Research 2009 37 1 13 10.1093/nar/gkn923 19033363 PMC2615629

[B17] Nam D Kim SY Gene-set approach for expression pattern analysis Brief Bioinform 2008 9 189 197 10.1093/bib/bbn001 18202032

