# Supplementary material for: Microarray Я US: a user-friendly graphical interface to Bioconductor tools that enables accurate microarray data analysis and expedites comprehensive functional analysis of microarray results
Source: BMC Res Notes. 2012 Jun 8;5:282. doi: 10.1186/1756-0500-5-282 (PMC3459790; doi:10.1186/1756-0500-5-282)
Supplement: Additional file 1 — List of the implemented Bioconductor packages. Complete list of the implemented Bioconductor packages with brief descriptions and references. [file 1756-0500-5-282-S1.pdf]

Additional file 1. List of the implemented Bioconductor packages

PUBLIC DATA ACCESS PACKAGES

- **GEOquery** ---- Get data from NCBI Gene Expression Omnibus (GEO) [1]
- **Geometadb** ---- A compilation of metadata from NCBI GEO [2]
- **ArrayExpress** ---- Access the ArrayExpress Microarray Database at EBI and build Bioconductor data structures [3]

PREPROCEESING AND NORMALIZATION PACKAGES

- **affy** ---- Methods for processing Affymetrix oligonucleotide arrays [4]
- **lumi** ---- Methods for processing Illumina BeadArrays [5-7]
- **vsn** ---- Variance stabilization and calibration for microarray data [8]
- **gcrma** ---- Background adjustment method using sequence information [9]

QUALITY CONTROL PACKAGES

- **arrayQualityMetrics** ---- Quality metrics on ExpressionSets [10]
- **affyQCReport** ---- QC Report Generation for affyBatch objects [11]
- **made4** ---- Multivariate analysis of microarray data using ADE4 [12]

DIFFERENTIALLY EXPRESSED GENE DETECTION PAKCAKGES

- **limma** ---- Linear models for microarray data [13]
- **siggenes** ---- SAM and Efron's empirical Bayes approaches [14]
- **RankProd** ---- Rank product method for identifying differentially expressed genes with application in meta-analysis [15]
- **maSigPro** ---- Microarray significant gene expression profile – find differences in time course data [16]

POWER ANALYSIS PACKAGES

- **ssize** ---- Estimate microarray sample size [17]

References

1. Sean D, Meltzer PS: **GEOquery: a bridge between the Gene Expression Omnibus (GEO) and BioConductor.** *Bioinformatics (Oxford, England)* 2007, **23:**1846-1847.
2. Zhu Y, Davis S, Stephens R, Meltzer PS, Chen Y: **GEOmetadb: powerful alternative search engine for the Gene Expression Omnibus.** *Bioinformatics (Oxford, England)* 2008, **24:**2798-2800.
3. Kauffmann A, Rayner TF, Parkinson H, Kapushesky M, Lukk M, Brazma A, Huber W: **Importing ArrayExpress datasets into R/Bioconductor.** *Bioinformatics (Oxford, England)* 2009, **25:**2092-2094.
4. Gautier L, Cope L, Bolstad BM, Irizarry RA: **affy--analysis of Affymetrix GeneChip data at the probe level.** *Bioinformatics (Oxford, England)* 2004, **20:**307-315.
5. Du P, Kibbe WA, Lin SM: **nuID: a universal naming scheme of oligonucleotides for illumina, affymetrix, and other microarrays.** *Biology direct* 2007, **2:**16.
6. Lin SM, Du P, Huber W, Kibbe WA: **Model-based variance-stabilizing transformation for Illumina microarray data.** *Nucleic acids research* 2008, **36:**e11.
7. Du P, Kibbe WA, Lin SM: **lumi: a pipeline for processing Illumina microarray.** *Bioinformatics (Oxford, England)* 2008, **24:**1547-1548.
8. Huber W, von Heydebreck A, Sultmann H, Poustka A, Vingron M: **Variance stabilization applied to microarray data calibration and to the quantification of differential expression.** *Bioinformatics (Oxford, England)* 2002, **18 Suppl 1:**S96-104.
9. Wu Z, Irizarry R, MacDonald J, Gentry J: **gcrma: Background Adjustment Using Sequence Information.** *R package version 2200* 2002.
10. Kauffmann A, Gentleman R, Huber W: **arrayQualityMetrics--a bioconductor package for quality assessment of microarray data.** *Bioinformatics (Oxford, England)* 2009, **25:**415-416.
11. Parman C, Halling C, Gentleman R: **affyQCReport: QC Report Generation for affyBatch objects.** *R package version 1260* 2010.
12. Culhane AC, Thioulouse J, Perriere G, Higgins DG: **MADE4: an R package for multivariate analysis of gene expression data.** *Bioinformatics (Oxford, England)* 2005, **21:**2789-2790.
13. Smyth GK: **Linear models and empirical bayes methods for assessing differential expression in microarray experiments.** *Statistical applications in genetics and molecular biology* 2004, **3:**Article3.
14. Schwender H: **siggenes: Multiple testing using SAM and Efron's empirical Bayes approaches.** *R package version 1220* 2009.
15. Hong F, Breitling R, McEntee CW, Wittner BS, Nemhauser JL, Chory J: **RankProd: a bioconductor package for detecting differentially expressed genes in meta-analysis.** *Bioinformatics (Oxford, England)* 2006, **22:**2825-2827.
16. Conesa A, Nueda MJ, Ferrer A, Talon M: **maSigPro: a method to identify significantly differential expression profiles in time-course microarray experiments.** *Bioinformatics (Oxford, England)* 2006, **22:**1096-1102.
17. Warnes GR, Liu P, Li F: **ssize: Estimate Microarray Sample Size.** *R package version 1220* 2009.
