# Supplementary material for: Microarray Я US: a user-friendly graphical interface to Bioconductor tools that enables accurate microarray data analysis and expedites comprehensive functional analysis of microarray results
Source: BMC Res Notes. 2012 Jun 8;5:282. doi: 10.1186/1756-0500-5-282 (PMC3459790; doi:10.1186/1756-0500-5-282)
Supplement: Additional file 2 — List of the supported functional analysis software. Description: Complete list of the supported functional analysis software for the Gene List Output Utility Tool. Access information, methods, input file requirements, supported organisms, matching Microarray Я US output file, and other details are listed for each supported software. [file 1756-0500-5-282-S2.pdf]

Additional file 2. List of the supported functional analysis software

Abbreviations:

DEG:	differentially expressed genes
FC:	fold change
FDR:	False Discovery Rate
P:	p value
SEA:	singular enrichment analysis
GSEA:	gene set enrichment analysis
MEA:	modular enrichment analysis

(For definitions on SEA, GSEA and MEA, please refer to Huang et al. 2009a [1]).

A. COMPREHENSIVE FUNCTIONAL PROFILING SOFTWARE (COMMERCIAL)

A.1 INGENUITY PATHWAY ANALYSIS (IPA)

**Web Site:**	http://www.ingenuity.com/
**Required ID:**	All major IDs accepted, allows multiple ID columns
**Required data type:**	DEG list with Probe ID or Gene Symbol, FC, p, FDR-adjusted p value
**Supported organisms:**	Human, mouse, rat; ortholog gene mapping for other major model organisms
**Required input file format:**	Tab delimitated .txt file
**Microarray Я US output file:**	your_file_name.IPA.txt

Functional analysis type:	SEA (supports multiple DEG lists comparison)
Content type:	Mixed (Human curated and computational predicted)
Major functional analysis categories:	Pathways, networks, miRNA targets, disease, biological functions and process, subcellular locations, and literatures.

A.2. NEXTBIO

**Web Site:**	http://www.nextbio.com/
**Required ID:**	All major IDs accepted, allows multiple ID columns
**Required data type:**	DEG list—Probe ID or Gene Symbol, along with FC, p, and FDR-adjusted p
**Supported organisms:**	Many major model organisms
**Required input file format:**	Tab delimitated .txt file
**Microarray Я US output file:**	your_file_name.NextBio.txt

Functional analysis type:	SEA (supports multiple DEG lists comparison)

Content type: Mixed (Human curated and computational predicted)
Major functional analysis categories: Pathways, GO, TF targets, miRNA targets, disease, protein domains, SNP, chromosomal locations, literatures. Also allows meta-analysis against pre-analyzed gene signatures from public high-throughput genomic studies

B. COMPREHENSIVE FUNCTIONAL PROFILING SOFTWARE (OPEN ACCESS)

B.1 NIH DAVID [1, 2]

Web Site: http://david.abcc.ncifcrf.gov/
Required ID: Probe set ID (for all standard CDF Affymetrix and Illumina arrays)
Required data type: DEG list-Probe set ID only
Supported organisms: Many major model organisms
Required input file format: Tab delimitated .txt file
Microarray Я US output file: your_file_name.DAVID.txt

Required ID: Gene symbol (for all custom CDF Affymetrix arrays)
Required data type: (1) DEG list-with Gene Symbol only
(2). Background list (only for data processed with a customized CDF)
Supported organisms: Many major model organisms
Required input file format: Tab delimitated .txt file
Microarray Я US output file: your_file_name.CDF-DAVID.txt

Functional analysis type: SEA and MEA
Content type: Mixed (Human curated and computational predicted)
Major functional analysis categories: Pathways (multiple database), GO, TF targets, miRNA targets, disease, protein domains, protein-protein interactions (multiple databases), GWAS, chromosomal locations, literature

B.2 GENETRAIL—ADVANCED GENE SET ENRICHMENT ANALYSIS [3]

Web Site: http://genetrail.bioinf.uni-sb.de/

SEA Mode
Required ID: Gene Symbol
Required data type: (1)DEG list-with Gene Symbol only
(2) Reference list (optional, only for data processed with a customized CDF)
Supported organisms: Many major model organisms
Required input file format: Tab delimitated .txt file
Microarray Я US output file: your_file_name.Genetrail-SEA.txt

GSEA mode

Required ID: Gene Symbol

Required data type: (1) The complete variance analyzed gene list-with gene symbol only, ranked by p
(2) Reference list (optional, only for data processed with a customized CDF)

Supported organisms: Many major model organisms

Required input file format: Tab delimitated .txt file

Microarray Я US output file: your_file_name.Genetrail-GSEA.txt

Functional analysis type: SEA and GSEA

Content type: Mixed (Human curated and computational predicted)

Major functional analysis categories: Pathways, GO, TF targets, miRNA targets, disease, protein domains, SNP, chromosomal locations

B.3 GENECODIS: INTERPRETING GENE LISTS THROUGH ENRICHMENT ANALYSIS AND INTEGRATION OF DIVERSE BIOLOGICAL INFORMATION [4]

Web Site: **http://genecodis.dacya.ucm.es/**

Required ID: Gene Symbol

Required data type: (1) DEG list-Gene Symbol only
(2) Reference list (optional, only for data processed with a customized CDF)

Supported organisms: Many major model organisms

Required input file format: Tab delimitated txt file.

Microarray Я US output file: your_file_name.GeneCodis.txt

Functional analysis type: SEA and MEA

Content type: Mixed (Human curated and computational predicted)

Major functional analysis categories: Pathways, GO (different levels and GOSlim), TF targets, miRNA targets, protein motifs

B.4 WEBGESTALT: AN INTEGRATED SYSTEM FOR EXPLORING GENE SETS IN VARIOUS BIOLOGICAL CONTEXTS [5]

Web Site: **http://bioinfo.vanderbilt.edu/wg_gsat/**

Required ID: Gene Symbol

Required data type: (1) DEG list—Gene Symbol with FC

Supported organisms: Many major model organisms

Required input file format: Tab delimitated txt file.

Microarray Я US output file: your_file_name_WebGestalt.txt

Functional analysis type: SEA

Content type: Mixed (Human curated and computational predicted)

Major functional analysis categories: Pathways (multiple database), GO, TF targets, miRNA targets, protein-protein interaction, chromosomal locations

Note: **Its GO analysis module is from the popular GOTM**

B.5 FATIGO +: A FUNCTIONAL PROFILING TOOL FOR GENOMIC DATA. INTEGRATION OF FUNCTIONAL ANNOTATION, REGULATORY MOTIFS AND INTERACTION DATA WITH MICROARRAY EXPERIMENTS [6]

**Web Site:**	**http://babelomics.bioinfo.cipf.es/functional.html**
**Required ID:**	Gene Symbol
**Required data type:**	DEG list-Gene symbol only
**Supported organisms:**	Many major model organisms
**Required input file format:**	Tab delimitated .txt file
**Microarray Я US output file:**	your_file_name.FatiGO.txt
**Web Site:**	http://babelomics.bioinfo.cipf.es/functional.html

Functional analysis type:	SEA
Content type:	Mixed (Human curated and computational predicted)
Major functional analysis categories:	Pathways, GO, GOSlim, TF targets, regulatory sequences; miRNA targets, protein domains;
Note:	Allows customized level setting for GO analysis.

B.6 TOPPCLUSTER: A MULTIPLE GENE LIST FEATURE ANALYZER FOR COMPARATIVE ENRICHMENT CLUSTERING AND NETWORK-BASED DISSECTION OF BIOLOGICAL SYSTEMS [7]

**Web Site:**	**http://toppcluster.cchmc.org/**
**Required ID:**	Gene Symbol
**Required data type:**	DEG list-symbol only
**Supported organisms:**	**mainly human, mouse and rat also workable**
**Required input file format:**	Tab delimitated .txt file
**Microarray Я US output file:**	your_file_name.ToppCluster.txt

Functional analysis type:	SEA
Content type:	Mixed (Human curated and computational predicted)
Major functional analysis categories:	Pathways, GO, TF targets, miRNA targets, disease, protein domains; protein-protein interaction, drugs, human/mouse phenotypes; co-expression gene sets; chromosomal locations, literatures
Note:	Allows multiple gene lists comparison

B.7 GSEA-P: A DESKTOP APPLICATION FOR GENE SET ENRICHMENT ANALYSIS [8]

**Web Site:**	**http://www.broad.mit.edu/GSEA**
**Required ID:**	All major IDs accepted, allows multiple ID columns

Required data type: (1) .GCT file of preprocessed data—Gene Symbol, natural scale intensity data for each sample
(2) .CLS file of phenotype labels
(3). RNK file of a variance analyzed completed gene list---Gene Symbol only, pre-ranked based on p value
Supported organisms: Many major model organisms
Required input file format: Tab delimitated files saved in .gct, .cls, .rnk format
Microarray Я US output file: your_file_name.GSEA.gct
your_file_name.GSEA.cls
your_file_name.GSEA.rnk

Note: GSEA requires either one .rnk file OR both .gct and .cls files for the analysis

Functional analysis type: GSEA
Content type: Mixed (Human curated and computational predicted)
Major functional analysis categories: Pathways, GO, TF targets, miRNA targets, various expression gene sets; chromosomal locations

C. TRANSCRIPTION FACTORS TARGETS ANALYSIS SOFTWARE

C.1 TRANSFIND—PREDICTING TRANSCRIPTIONAL REGULATORS FOR GENE SETS [9]

Web Site: http://transfind.sys-bio.net/
Required ID: Gene Symbol
Required data type: (1) DEG list—Gene Symbol only
(2) Reference list (optional, only for data processed with a customized CDF)
Supported organisms: Many major model organisms
Required input file format: Tab delimitated .txt file
Microarray Я US output file: your_file_name.TransFind.txt

Functional analysis type: SEA
Content type: Mixed (Human curated and computational predicted)
Major functional analysis categories: Transcription factors with conserved binding motif

C.2 TFACTS—TRANSCRIPTION FACTOR REGULATION CAN BE ACCURATELY PREDICTED FROM THE PRESENCE OF TARGET GENE SIGNATURES IN MICROARRAY GENE EXPRESSION DATA [10]

Web Site: http://www.tfacts.org/
Required ID: Gene Symbol
Required data type: (1) Up-regulated DEG list-symbol only
(2) Down-regulated DEG list-symbol only

Supported organisms: Primarily human, but also mouse/rat human ortholog gene
Required input file format: Tab delimitated txt file.
Microarray Я US output file: your_file_name.UP_TFactS.txt; your_file_name.DOWN_TFactS.txt

Functional analysis type: SEA
Content type: Human curated
Major functional analysis categories: Transcription factors regulation (sign-sensitive)

D. PATHWAY ONLY ANALYSIS SOFTWARE

D.1 ONTO-TOOLS PATHWAY-EXPRESS [11]

Web Site: **http://vortex.cs.wayne.edu/projects.htm**
Required ID: Gene Symbol
Required data type: (1) DEG list—Gene Symbol with FC
(2) Reference list (optional, only for data processed with a customized CDF)
Supported organisms: Many major model organisms
Required input file format: Tab delimitated txt file.
Microarray Я US output file: your_file_name.Onto-PE.txt

Functional analysis type: SEA
Content type: Human curated
Major functional analysis categories: Pathways with impact factors (calculated based on the gene expression directions and the topography of a pathway).

D.2 GENMAPP 2 [12]

Web Site: **http://www.genmapp.org/**
Required ID: Ensembl Symbol
Required data type: (1) The complete variance analyzed gene list Ensembl ID, FC and p
(2) Reference list (optional, only for data processed with a customized CDF)
Supported organisms: Many major model organisms
Required input file format: Tab delimitated txt file.
Microarray Я US output file: your_file_name.GenMAPP.txt

Functional analysis type: SEA
Content type: Human curated
Major functional analysis categories: Pathways

E. EXPRESSION GENE SIGNATURES SEARCH SOFTWARE

E.1 EXALT-- WEB-BASED INTERROGATION OF GENE EXPRESSION SIGNATURES USING EXALT [13]

Web Site: http://seq.mc.vanderbilt.edu/exalt/
Required ID: Probe ID and Gene Symbol
Required data type: Preprocessed data (natural scale) with all samples listed
Required experiment type: One factor with up to 9 levels, with at least 2 replicates in each group
Supported organisms: Human, mouse, rat
Required input file format: Tab delimitated .txt file
Microarray Я US output file: your_file_name_EXALT.txt

Functional analysis type: General expression signatures mining
Content type: Human curated
Major functional analysis categories: Public expression data signatures

E.2 THE CONNECTIVITY MAP: USING GENE-EXPRESSION SIGNATURES TO CONNECT SMALL MOLECULES, GENES, AND DISEASE [14]

Web Site: http://www.broadinstitute.org/cmap/
Required ID: Affymetrix HG-U133A probe set ID, mapped from Gene Symbol
Required data type: (1) Up-regulated DEG list- Affymetrix HG-U133A probe set ID only
(2) Down-regulated DEG list- Affymetrix HG-U133A probe set ID only
Supported organisms: Primarily human, but also mouse/rat human ortholog genes
Required input file format: Tab delimitated txt file.
Microarray Я US output file: your_file_name.UP_CMAP.grp; your_file_name.DOWN_CMAP.grp
Note: Total DEG list should not exceed 1000 genes.

Functional analysis type: Expression signatures mining
Content type: Human curated
Major functional analysis categories: Cell-line drug treatment expression signatures

F. GENE ONTOLOGY (GO) ONLY ANALYSIS TOOL

F.1 GORILLA—A TOOL FOR DISCOVERY AND VISUALIZATION OF ENRICHED GO TERMS IN RANKED GENE LISTS [15]

Web Site: http://cbl-gorilla.cs.technion.ac.il/
Required ID: Gene Symbol
Required data type: The complete variance analyzed gene list-with gene symbol only, ranked by p
Supported organisms: Many major model organisms

Required input file format: Tab delimitated txt file.
Microarray Я US output file: your_file_name.GOrilla.txt

Functional analysis type: SEA and GSEA
Content type: Mixed (Human curated and computational predicted)
Major functional analysis categories: GO

F.2 FUNCASSOCIATE 2.0-- NEXT GENERATION SOFTWARE FOR FUNCTIONAL TREND ANALYSIS [16]

Web Site: http://llama.med.harvard.edu/funcassociate/
Required ID: Gene Symbol
Required data type: DEG list--Gene Symbol only
Supported organisms: Many major model organisms
Required input file format: Tab delimitated txt file.
Output file name: your_file_name.FuncAssociate.txt

Functional analysis type: SEA
Content type: Mixed (Human curated and computational predicted)
Major functional analysis categories: GO
Note: Allows customized GO Evidence Codes setting

F.3 GOMINER (HIGH-THROUGHPUT)—AN INTEGRATIVE GENE ONTOLOGY TOOL FOR INTERPRETATION OF MULTIPLE-MICROARRAY EXPERIMENTS [17]

Web Site: http://discover.nci.nih.gov/gominer/htgm.jsp
Required ID: Gene Symbol
Required data type: DEG list(s)--with Gene Symbol with + or - signs for up/down expression change directions
Supported organisms: Many major model organisms
Required input file format: Tab delimitated txt file.
Output file name: your_file_name.GoMiner.txt

Functional analysis type: SEA (Supports multiple DEG lists comparison)
Content type: Mixed (Human curated and computational predicted)
Major functional analysis categories: GO

G. MIRNA AND MRNA INTEGRATED ANALYSIS

G.1 MAGIA—A WEB-BASED TOOL FOR MIRNA AND GENES INTEGRATED ANALYSIS [18]

Web Site: http://gencomp.bio.unipd.it/magia
Required ID: Entrez ID
Required data type: DEG list—Entrez ID, preprocessed natural scale intensities data for each sample
Supported organisms: Human only

Required input file format: Tab delimitated txt file.
Microarray Я US output file: your_file_name.MAGIA.txt
Web site: http://gencomp.bio.unipd.it/magia/start/

Functional analysis type: Correlation
Content type: Mixed (Human curated and computational)
Major functional analysis categories: miRNA targets prediction, miRNA-mRNA expression correlation analysis
Note: Sample names and order must be matched between the mRNA and miRNA lists.

G.2 MMIA : MIRNA AND MRNA INTEGRATED ANALYSIS [19]

Web Site: **http://156.56.93.156/~MMIA/index.html**
Required ID: Gene Symbol
Required data type: Preprocessed data—Gene Symbol and preprocessed linear intensities data for each sample
Supported organisms: Human only
Required input file format: Tab delimitated txt file.
Microarray Я US output file: your_file_name.MAGIA.txt

Functional analysis type: GSEA and Correlation
Content type: Mixed (Human curated and computational)
Major functional analysis categories: miRNA targets prediction, miRNA-mRNA expression correlation analysis; TFBS in miRNA promoter; diseases; pathways, GO, cancer gene sets, chromosomal locations
Note: sample name and order must be matched between the mRNA and miRNA lists.

G.3 GENESET2MIRNA [20]

Web Site: **http://mips.helmholtz-muenchen.de/proj/gene2mir/**
Required ID: Gene Symbol
Required data type: DEG list—Gene Symbol only
Supported organisms: Human, mouse, rat
Required input file format: Tab delimitated txt file.
Microarray Я US output file: your_file_name.MAGIA.txt

Functional analysis type: Correlation
Content type: Mixed (Human curated and computational)
Major functional analysis categories: miRNA targets prediction, miRNA-mRNA expression correlation analysis
Note: sample name and order must be matched between the mRNA and miRNA lists.

H. OTHER TOOLS

H.1 GENEPATTERN--USING GENEPATTERN FOR GENE EXPRESSION ANALYSIS [21]

Web Site:

www.broadinstitute.org/cancer/software/genepattern/

**Required ID:**	All major IDs accepted, allows multiple ID columns
**Required data type:**	(1) GCT file of preprocessed data—Gene Symbol, natural scale intensity data for each sample<br>(2) CLS file of phenotype labels
**Supported organisms:**	Many major model organisms
**Required input file format:**	Tab delimitated file saved with .gct or .cls
**Microarray Я US output file:**	your_file_name.GenePattern.gct<br>your_file_name.GenePattern.cls
Analysis type:	Statistical and visual analysis of microarray data
Note:	Over 100 programs available in GenePattern for a wide spectrum of microarray data analysis and manipulation

References

1. Huang DW, Sherman BT, Lempicki RA: **Systematic and integrative analysis of large gene lists using DAVID bioinformatics resources.** *Nat Protoc* 2009, **4:**44-57.
2. Huang DW, Sherman BT, Lempicki RA: **Bioinformatics enrichment tools: paths toward the comprehensive functional analysis of large gene lists.** *Nucleic Acids Res* 2009, **37:**1-13.
3. Backes C, Keller A, Kuentzer J, Kneissl B, Comtesse N, Elnakady YA, Muller R, Meese E, Lenhof HP: **GeneTrail--advanced gene set enrichment analysis.** *Nucleic Acids Res* 2007, **35:**W186-192.
4. Nogales-Cadenas R, Carmona-Saez P, Vazquez M, Vicente C, Yang X, Tirado F, Carazo JM, Pascual-Montano A: **GeneCodis: interpreting gene lists through enrichment analysis and integration of diverse biological information.** *Nucleic Acids Res* 2009, **37:**W317-322.
5. Zhang B, Kirov S, Snoddy J: **WebGestalt: an integrated system for exploring gene sets in various biological contexts.** *Nucleic Acids Res* 2005, **33:**W741-748.
6. Al-Shahrour F, Minguez P, Tarraga J, Medina I, Alloza E, Montaner D, Dopazo J: **FatiGO +: a functional profiling tool for genomic data. Integration of functional annotation, regulatory motifs and interaction data with microarray experiments.** *Nucleic Acids Res* 2007, **35:**W91-96.
7. Kaimal V, Bardes EE, Tabar SC, Jegga AG, Aronow BJ: **ToppCluster: a multiple gene list feature analyzer for comparative enrichment clustering and network-based dissection of biological systems.** *Nucleic Acids Res* 2010, **38:**W96-102.
8. Subramanian A, Kuehn H, Gould J, Tamayo P, Mesirov JP: **GSEA-P: a desktop application for Gene Set Enrichment Analysis.** *Bioinformatics* 2007, **23:**3251-3253.
9. Kielbasa SM, Klein H, Roider HG, Vingron M, Bluthgen N: **TransFind--predicting transcriptional regulators for gene sets.** *Nucleic Acids Res* 2010, **38:**W275-280.
10. Essaghir A, Toffalini F, Knoops L, Kallin A, van Helden J, Demoulin JB: **Transcription factor regulation can be accurately predicted from the presence of target gene signatures in microarray gene expression data.** *Nucleic Acids Res* 2010, **38:**e120.
11. Draghici S, Khatri P, Tarca AL, Amin K, Done A, Voichita C, Georgescu C, Romero R: **A systems biology approach for pathway level analysis.** *Genome Res* 2007, **17:**1537-1545.
12. Salomonis N, Hanspers K, Zambon AC, Vranizan K, Lawlor SC, Dahlquist KD, Doniger SW, Stuart J, Conklin BR, Pico AR: **GenMAPP 2: new features and resources for pathway analysis.** *BMC Bioinformatics* 2007, **8:**217.
13. Wu J, Qiu Q, Xie L, Fullerton J, Yu J, Shyr Y, George AL, Jr., Yi Y: **Web-based interrogation of gene expression signatures using EXALT.** *BMC Bioinformatics* 2009, **10:**420.
14. Lamb J: **The Connectivity Map: a new tool for biomedical research.** *Nat Rev Cancer* 2007, **7:**54-60.
15. Eden E, Navon R, Steinfeld I, Lipson D, Yakhini Z: **GOrilla: a tool for discovery and visualization of enriched GO terms in ranked gene lists.** *BMC Bioinformatics* 2009, **10:**48.
16. Berriz GF, Beaver JE, Cenik C, Tasan M, Roth FP: **Next generation software for functional trend analysis.** *Bioinformatics* 2009, **25:**3043-3044.
17. Zeeberg BR, Feng W, Wang G, Wang MD, Fojo AT, Sunshine M, Narasimhan S, Kane DW, Reinhold WC, Lababidi S, et al: **GoMiner: a resource for biological interpretation of genomic and proteomic data.** *Genome Biol* 2003, **4:**R28.
18. Sales G, Coppe A, Bisognin A, Biasiolo M, Bortoluzzi S, Romualdi C: **MAGIA, a web-based tool for miRNA and Genes Integrated Analysis.** *Nucleic Acids Res* 2010, **38:**W352-359.

19. Nam S, Li M, Choi K, Balch C, Kim S, Nephew KP: **MicroRNA and mRNA integrated analysis (MMIA): a web tool for examining biological functions of microRNA expression.** *Nucleic Acids Res* 2009, **37:**W356-362.
20. Antonov AV, Dietmann S, Wong P, Lutter D, Mewes HW: **GeneSet2miRNA: finding the signature of cooperative miRNA activities in the gene lists.** *Nucleic Acids Res* 2009, **37:**W323-328.
21. Kuehn H, Liberzon A, Reich M, Mesirov JP: **Using GenePattern for gene expression analysis.** *Current protocols in bioinformatics / editoral board, Andreas D Baxevanis [et al* 2008, **Chapter 7:**Unit 7 12.
