# Supplementary material for: Microarray Я US: a user-friendly graphical interface to Bioconductor tools that enables accurate microarray data analysis and expedites comprehensive functional analysis of microarray results
Source: BMC Res Notes. 2012 Jun 8;5:282. doi: 10.1186/1756-0500-5-282 (PMC3459790; doi:10.1186/1756-0500-5-282)
Supplement: Additional file 3 — List of the supported microarray data types. Complete list of the supported microarray chips. [file 1756-0500-5-282-S3.pdf]

Additional file 3. List of the supported microarray data types

**Affymetrix Intensity Data (.CEL files)**	
**Human**	HG-U133A; HG-U133A_2; HG-U133B; HG-U133_Plus_2; HG_U95A; HG_U95Av2; HG_Focus
**Mouse**	MG_U74Av2; MG_U74Bv2; MG_U74Cv2; Mouse430_2; Mouse430A_2; MOE430A; MOE430B
**Rat**	RG_U34A; Rat230_2; RAE230A; RAE230B
**Illumina Expression Beadchips (GenomeStudio/BeadStudio outputs)**	
**Human** (version 1 to 3)	HumanWG-6; HumanRef-8; HumanHT-12
**Mouse** (version 1 to 2)	MouseWG-6; MouseRef-8
**Rat** (version 1)	RatRef-12
